# Perceptual Bias in Motion Discrimination is Related to Asymmetric Interhemispheric Alpha Traveling Waves

**DOI:** 10.1002/advs.202414623

**Published:** 2025-08-30

**Authors:** Luca Tarasi, Andrea Alamia, Vincenzo Romei

**Affiliations:** ^1^ Dipartimento di Psicologia Università di Bologna and Centro studi e ricerche in Neuroscienze Cognitive Università di Bologna Cesena Italy; ^2^ Centre de Recherche Cerveau et Cognition (CerCo) CNRS Université de Toulouse Toulouse 31052 France; ^3^ Universidad Antonio de Nebrija Madrid 28015 Spain

**Keywords:** BRAIN OSCILLATIONS, EEG, INTER‐HEMISPHERIC CONNECTIVITY, motion perception, traveling waves

## Abstract

Perception relies on hierarchical processes integrating sensory data into higher‐order models about the world. In the sensory domain, this hierarchy also involves horizontal pathways aiding interhemispheric interactions. For example, recent focus on the V5‐V5 network revealed its role in motion processing. However, despite converging evidence demonstrating the crucial role of oscillatory activity in inter‐areas communication, the specific rhythmic code governing V5‐V5 interaction is unclear. Through traveling wave analysis, this gap is filled by identifying the alpha/low‐beta frequency (9–16 Hz) as crucial for long‐range, interhemispheric communication between V5 regions. Notably, a pronounced imbalance is observed: stronger waves travelled in the left‐to‐right‐V5 direction. Crucially, this observation extends to the behavioural level, as Signal Detection Theory analysis uncovered a correlation between the highlighted imbalance and a perceptual bias favouring leftward motion reporting. Our findings underscore asymmetrical projections between V5 regions, highlighting the interplay between inter‐hemispheric traveling wave patterns in dictating motion perception.

## Graphical abstract

The study shows an asymmetric connectivity between visual motion areas, with alpha travelling waves favouring left‐to‐right (thick red arrow) over right‐to‐left (thin blue arrow) communication. This asymmetry predicts a bias toward reporting leftward (higher number of red dots) over rightward (lower number of blue dots) motion direction, regardless of the actual movement (equal number of left/right black arrows).

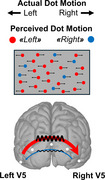

## 1 Introduction

The visual brain system operates on a hierarchical basis.^[^ ^1^ ^]^ This hierarchy extends beyond the classical bottom‐up and top‐down flow, involving horizontal pathways emerging through the interaction between homologous areas of the two hemispheres via callosal connections.^[^ ^2^, ^3^, ^4^ ^]^ These interhemispheric interactions play a crucial role across various cognitive and perceptual domains, including attention, language, and motor control.^[^ ^5^, ^6^, ^7^ ^]^ Recently, a cortico‐cortical paired associative stimulation (ccPAS) study^[^ ^8^ ^]^ has characterized the horizontal interactions within the V5‐V5 system using the apparent motion quartet paradigm,^[^ ^9^ ^]^ revealing functional dissociations between interhemispheric visual networks. Employing a fine‐tuned ccPAS protocol (for a review see^[^ ^10^ ^]^), the authors specifically enhanced connectivity between the V5 regions of the left and right hemispheres. Their findings showcased that reinforcing the left‐to‐right V5 pathway heightened sensitivity to horizontal visual motion, whereas strengthening the right‐to‐left V5 pathway was found to be less effective in producing a similar effect. This asymmetry was further reflected at the behavioural level, with participants exhibiting a tendency toward perceiving leftward motion in an apparent motion direction task^8^. This evidence converged with previous studies showing that the strength of structural connectivity in the V5‐V5 pathway is associated with enhanced horizontal motion sensitivity in the motion quartet task.^[^ ^11^ ^]^

However, the participant's response, both in the motion quartet and apparent motion direction tasks, is subjective due to the illusory nature of the induced percepts. Therefore, in principle it is not feasible to determine whether the highlighted tendency to report a leftward movement was due to better motion sampling or rather to a response bias. Furthermore, there is no understanding of the underlying physiological mechanisms shaping these interhemispheric interactions.

**Figure 1:**
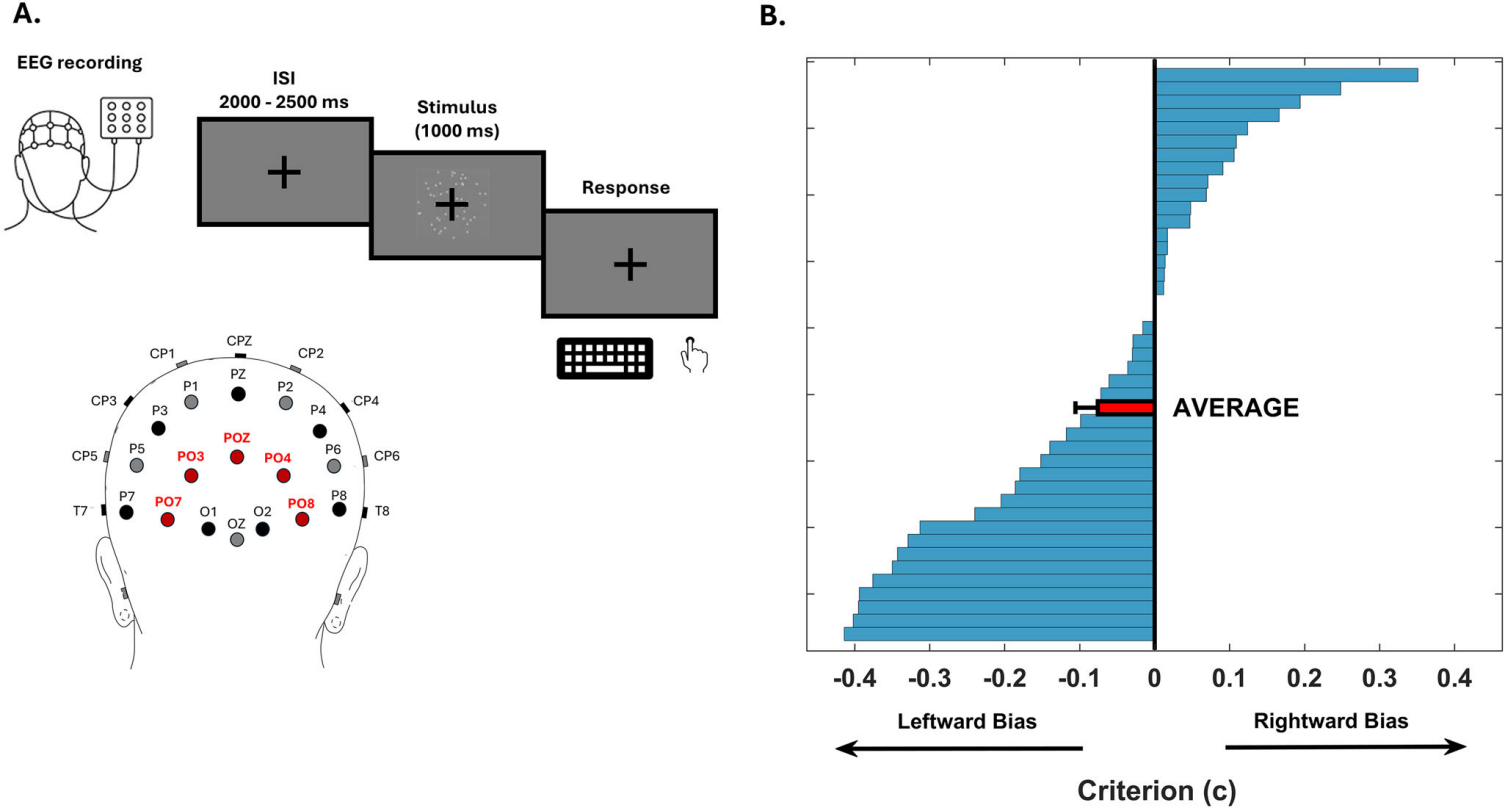
Experimental Design and Results: Perceptual Bias in Reporting Leftward Movement in a Motion Discrimination Task. A) 42 participants completed a random dot motion (RDM) discrimination task while their brain activity was recorded non‐invasively using EEG. Visual stimuli consisted of 400 white dots moving within a central square region on the screen, presented at a distance of 70 cm. The dots moved coherently either leftward or rightward for 1000 ms. The percentage of coherently moving dots was adjusted during a titration phase to achieve a discrimination accuracy of 75%. In the bottom panel, in red are depicted the electrodes used to compute the traveling waves. The waves traveling between PO7 and PO8 were calculated since these electrodes are the closest to the area involved in motion analysis (V5).^[^ ^16^, ^17^, ^18^ ^]^ B)Signal Detection Theory (SDT) indexcriterion was derived, which aids in tracking the individual response bias. The majority of the participants showed a preference for leftward motion perception (c = ‐0.076 ± 0.03, p = 0.015), as depicted in the average score (red bar). Each blue bar represents the individual bias shown by the participants.

To bridge this gap, 42 participants were tasked with completing a random dot motion (RDM) discrimination task while their brain activity was recorded non‐invasively using EEG (**Figure** **1**A). Through this protocol, Signal Detection Theory (SDT) indices of sensitivity and criterion^[^ ^12^ ^]^ were derived, which aid in disentangling objective signal sampling capability from response bias.

Through the experimental protocol setup, the study aimed to investigate: 1) the potential presence of response bias in reporting a specific direction of movement in the RDM, 2) the rhythmic code through which interaction in the V5‐V5 network occurs via analysis of oscillatory traveling waves, 3) any imbalance in traveling waves based on the directionality (i.e., left‐to‐right vs. right‐to‐left) of the connections, and 4) how the observed pattern in interhemispheric connectivity explains the behavioural data related to decisional bias. Based on previous evidence,^[^ ^8^, ^11^ ^]^ it was hypothesized that a) there is a direction‐specific imbalance in the strength of V5‐V5 connectivity, with information flows favouring the left‐to‐right pathway, b) these interactions would be underpinned by an oscillatory synchronization within the alpha‐beta band, as converging evidence has demonstrated that they play a crucial role in sensory information transferring and long‐range connectivity,^[^ ^13^, ^14^, ^15^ ^]^ c) the imbalances in effective connectivity underlie the decision bias in reporting left‐ vs. right‐ward movement.

## 2 Results

### 2.1 Human Observers Manifest a Leftward Bias in Motion Perception

42 participants completed a random dot motion discrimination task while brain activity was recorded non‐invasively using EEG. In the first phase of the study, participants completed a preparatory step in which the coherence of moving dots necessary for each individual to achieve 75% accuracy in the task was determined. The coherence threshold was defined as the minimum level of motion coherence necessary for participants to reliably perceive the direction of motion. This threshold was estimated individually for each participant, yielding a mean threshold across subjects of 7.39 ± 0.44% (mean ± sem). This subject‐specific threshold was then used in the main task, which comprised 216 trials (Figure 1A, top panel). Within each trial, the titrated percentage of dots could move either leftward or rightward for 1000ms, and participants were required to indicate the perceived direction of movement by pressing a key on the keyboard. By employing a Signal Detection Theory Model, the computational parameters of d' (task sensitivity) and criterion (decision bias) were extracted to highlight any response tendencies within the sample. The conducted behavioural analysis revealed a bias in motion discrimination among participants (Figure 1B; c = ‐0.076 ± 0.03, t_41_ = ‐2.52; p = 0.015; Figure 1), with participants showing a proclivity in reporting leftward movement. This finding is noteworthy as it demonstrates, for the first time, the presence of a systematic perceptual bias toward reporting leftward motion, even in contexts where real motion is presented but sensory evidence is weak—thereby extending beyond previously reported effects limited to illusory contexts,^[^ ^8^ ^]^ where motion perception arises solely from subjective interpretation. In fact, it persists even in situations where there is an objective motion direction, such as in the RDM task. Moreover, the mean d′ across participants was 1.31 ± 0.68 (mean ± sem), indicating that they performed the task correctly, as d′ was significantly above zero (i.e., change detection performance; t_41_ = 19.36, p < 0.01).

### 2.2 The Strength of Traveling Alpha–Beta Waves Exhibits an Interhemispheric Asymmetry

It was then investigated whether the strength of information exchange between the two motion‐sensitive regions varied depending on directional factors (i.e., left‐to‐right V5 vs. right‐to‐left V5), and whether this imbalance could account for the bias observed at the behavioural level. It was hypothesized that the flow of information between the two V5 areas would show greater strength in the left‐to‐right direction compared to the opposite direction (Figure 1).

In order to test this hypothesis, the analyses focused on how oscillatory dynamics propagated as traveling waves between the two brain areas.^[^ ^19^, ^20^ ^]^ Oscillatory traveling waves can be defined as patterns of brain activity that propagate across brain regions in a synchronized and rhythmic manner.^[^ ^21^, ^22^ ^]^ Therefore, traveling waves manifest as rhythmic fluctuations in voltage that travel across the scalp. These waves may reflect large‐scale coordination of neural activity and have been associated with various cognitive processes such as perception, attention, and memory.^[^ ^23^, ^24^, ^25^ ^]^ Importantly, traveling waves may serve as a way to quantify cortical connectivity. In particular, in this study, the amount of traveling waves were determined using an analysis based on a 2D‐FFT procedure, which quantifies the waves propagating in both directions of a line of electrodes (^[^ ^19^, ^23^, ^26^, ^27^, ^28^, ^29^ ^]^ see methods for details).

**Figure 2:**
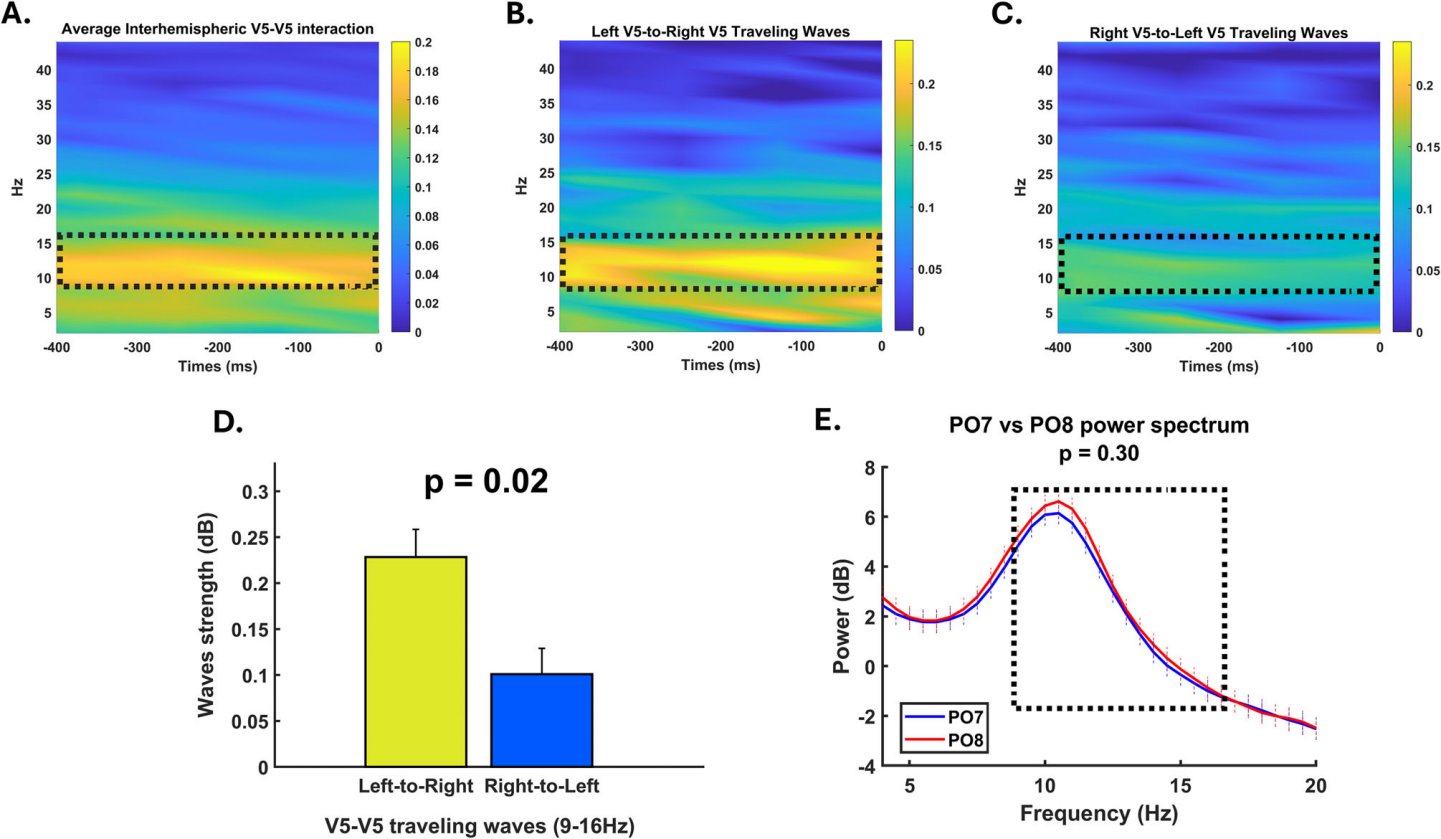
Stronger traveling waves flow in the left‐to‐right V5 direction. The figure describes the strength in dB of the traveling waves in the left V5‐right V5 circuit. A higher value implies a greater occurrence of traveling waves at the specific time point and frequency. The maps take into account both waves traveling in the left‐to‐right direction and right‐to‐left direction, as well as their average. A) The analysis reveals that pre‐stimulus interhemispheric interaction between the two V5 areas predominantly falls within the alpha/low‐beta range (9–16 Hz). This finding underscores the frequency specificity of interhemispheric communication in visual processing as the effect did not hold valid when considering the theta and high‐beta bands (all t_41_ < 1.04, all p >0.30, all BF < 0.28, – see Figure S3, Supporting Information). B) The strength of these traveling waves varies with the directionality of connections. Notably, neural information flow in the alpha/low‐beta bands is significantly stronger in the left‐to‐right direction compared to the right‐to‐left direction (t_41_ = 2.37, p = 0.02). Error bars indicate SEM. Asterisks denote significance levels (p < .01; two‐tailed paired t‐test). C) Conversely, the flow of information from left to right V5 is notably weaker. This asymmetry highlights the directional bias in interhemispheric information transfer. D) Histogram representation of the imbalance in interhemispheric traveling waves between V5s areas. E) The observed effect in the traveling waves is not due to a local mechanism related to the power difference between the two electrodes near the V5 areas (i.e., PO7 and PO8) (t_41_ = ‐1.05, p = 0.30, BF = 0.28). Thus, the effect appears to be specifically tied to the flow of information traveling along the V5‐V5 network.

First, the frequency band in which the interaction between the two visual motion areas takes place were examined. **Figure** **2**A–C shows the amount of traveling waves in dB (see methods for details) for all frequency bands over time. Our analysis revealed that the pre‐stimulus interhemispheric interaction between the two V5 areas occurs mainly in the alpha/low‐beta range (9–16 Hz). Subsequently, it was investigated whether there was a difference in the strength of these traveling waves when considering the directionality of the connections. Crucially, the flow of neural information in the alpha/low‐beta bands was much stronger when considering the left‐to‐right direction (left‐to‐right alpha‐beta wave strength [dB] = 0.23 ± 0.03) compared to the flow of information conveyed in the opposite direction (right‐to‐left alpha‐beta wave strength [dB] = 0.10 ± 0.03, t_41_ = 2.37, p = 0.02) (Figure 2B,C). This effect was specific to the visual system, as the same interhemispheric imbalance in traveling wave strength was not identified within the centro‐parietal and fronto‐frontal interhemispheric connections (Figure S1, Supporting Information). Furthermore, the effect was frequency‐specific because there was no difference in the traveling wave strength in the theta and high‐beta bands based on the directionality of the connections considered (all t_41_ < 1.04, all p >0.30, all BF < 0.28, Figure S3, Supporting Information).

Importantly, the highlighted results were corroborated by using two independent metrics of neural synchronization. First, the phase shift in the alpha–beta band between the homologous electrodes PO7 and PO8 were analysed. The Rayleigh test for non‐uniformity assessed whether the interhemispheric phase differences were consistently oriented across participants—an indicator of systematic directionality in the signal propagation. The test revealed a significant non‐uniform distribution (z = 31.08, p < 0.001), with a mean phase shift of 4.07 ± 4.68 degrees. This result indicates that the phase at PO7 consistently led that at PO8 across participants, which supports the idea that interhemispheric flow privileges the left‐to‐right direction. At the sensor level, computing the mean absolute phase shift yielded a temporal delay between the PO7 and PO8 activity of ≈4 ms at 10 Hz. This primary PO7‐to‐PO8 finding was further supported by demonstrating that PO7 also led each intermediate right‐hemisphere neighbour (PO3, POz, PO4). For each pair, the instantaneous alpha‐beta band phase difference showed a consistent positive mean direction across participants (all mean phase shift > 5.82 ± 1.76 degrees, all z > 28.90, all p < 0.01), indicating a stepwise left‐to‐right progression across posterior electrodes. Crucially, applying the same phase analysis in source space, the left V5 source led the right V5 source by a comparable positive phase angle (mean phase shift = 16.27 ± 7.49 degrees, z = 17.23, p < 0.01), mirroring the electrode‐level pattern and supporting a left‐initiated interhemispheric propagation of alpha activity. Moreover, at the source level, the mean absolute delay increased to ≈7 ms, reflecting slower apparent propagation when source‐reconstructed data were used. The very same pattern of results emerged when analyzing Granger causality as a measure of effective connectivity, further corroborating the presence of an imbalance in interhemispheric connectivity. Specifically, the flow from PO7 to PO8 (GC _PO7 to PO8_ = 0.063 ± 0.012) was stronger than the PO8‐to‐PO7 flow (GC _PO8 to PO7_ = 0.034 ± 0.004, t_40_ = 2.39, p = 0.02). Lastly, this sensor‐level analysis was validated at the source level, showing that the left V5 transmits more information in the alpha‐beta band to the right V5 (GC _lV5 to rV5_ = 0.053 ± 0.01) than in the opposite direction (GC _lV5 to lV5_ = 0.037 ± 0.007, t_40_ = 2.66, p = 0.01). Similarly, the same analysis performed on the rV1 and lV1 sources revealed no significant effects (GC _lV1 to rV1_ = 0.049 ± 0.008, GC _rV1 to lV1_ = 0.038 ± 0.007, t_39_ = 1.68, p = 0.11, BF = 0.61). This result highlights the spatial specificity of the effect to the two motion‐sensitive areas, suggesting that it does not generalize to other interhemispheric connections within the visual system.

### 2.3 Traveling Waves Asymmetry within the V5‐V5 Network Predicts Individual Perceptual Bias

**Figure 3:**
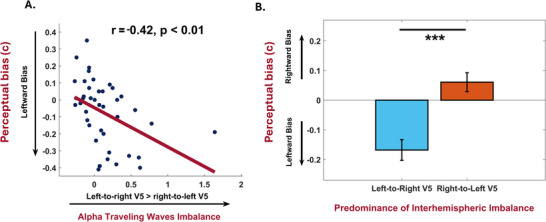
Perceptual bias in motion discrimination is determined by asymmetric interhemispheric alpha traveling waves. A) Correlation analysis between the imbalance in traveling waves between the two hemispheres and the decisional bias. The significant negative relationship (r _Pearson_ = – 0.42, p < 0.01) indicates that the stronger left‐to‐right V5 connections explain the observed leftward bias at the behavioural level. B) Analysis of the decisional criterion in the group with predominant left‐to‐right V5 connections and the group with predominant right‐to‐left V5 connections. The results indicate that the two groups exhibit significantly different decisional criteria (t_40_ = 4.58, p < 0.01), while maintaining the same level of sensitivity (t_40_ = 0.39, p = 0.70). While the left‐to‐right V5 predominant group shows a strong leftward bias, the group with predominant right‐to‐left traveling waves shows a rightward bias. Notably, the decisional bias is much stronger in the left‐to‐right group compared to the right‐to‐left group. Error bars indicate SEM. Asterisks denote significance levels (p < .01; two‐tailed paired t‐test).

Finally, it was examined whether the highlighted imbalance in hemispheric connectivity serves as a predictor for the magnitude of individual bias in motion perception. A significant positive association was unveiled between the inter‐individual difference in waves laterality and criterion (**Figure** **3**A; r _Pearson_ = ‐0.42, p < 0.01; r _Spearman_ = ‐0.51, p < 0.01). This suggests that the degree of imbalance in alpha/low‐beta V5 interhemispheric connectivity correlates with the strength of the leftward bias exhibited by participants—meaning that greater left V5‐to‐right V5 imbalance leads to a stronger bias. To ensure the reliability of our findings, robust correlations were conducted, which confirm the consistency of our initial analysis (r _Pearson skipped_ = ‐0.46, CI = [‐0.24; ‐0.64], r _Spearman skipped_ = ‐0.45, CI = [‐0.18; ‐0.67]). Furthermore, it was evaluated whether the identified effect might actually depend on a local effect, related to the activity of the electrodes closest to the areas that process motion information (i.e., PO7 and PO8). Control analyses demonstrated both that in the 9–16 Hz band the power in the two electrodes does not diverge (t_41_ = ‐1.05, p = 0.30, BF = 0.28; same pattern when comparing PO3 and PO4, t_41_ = ‐1.19, p = 0.24, BF = 0.32; see Figure S2, Supporting Information), and that the difference in power between the two sensors is not able to intercept the participants' decision bias (r _Pearson_ = 0.13, p = 0.41; r _Spearman_ = 0.10, p = 0.52; r _Pearson skipped_ = 0.12, CI = [‐0.46 0.24]; r _Spearman skipped_ = 0.10, CI = [‐0.44 0.24]).

Furthermore, V5‐V5 interhemispheric communication is not associated with sensitivity (d‘) in the task, highlighting the specificity of the effect (r _Pearson_ = ‐0.04, p = 0.77, BF = 0.12; r _Spearman_ = 0.002, p = 0.98). To corroborate this evidence, participants were divided into two groups based on whether they exhibited stronger traveling waves in the left‐to‐right or the right‐to‐left direction and compared their task performance. Although there were no significant differences in task sensitivity (d_left‐to‐right group_ = 1.29 ± 0.10, d_right‐to‐left group_ = 1.36 ± 0.09, t_40_ = 0.39, p = 0.70, BF = 0.32), decision criteria strongly varied between the two groups. Specifically, participants with a predominance of traveling waves from left to right showed a pronounced bias toward leftward motion (c = ‐0.16 ± 0.04). Conversely, the group characterized by stronger connections from right to left displayed a rightward decisional bias (c = 0.04 ± 0.03, t_40_ = 4.58, p < 0.01). As a confirmatory analysis, it was demonstrated that the group showing an over‐communication from the left to the right V5 areas exhibited a bias different from zero (where a criterion of 0 indicates no bias; t_24_ = 4.80, p < 0.01). Additionally, the group with predominant connectivity from the right to the left V5 exhibited a rightward bias that was also significantly different from zero (t_16_ = 1.90, p = 0.037). Thus, once again, it was shown that the predominance of interhemispheric imbalance between the two motion areas exerts a stronger influence on determining the direction of the perceptual bias.

## 3 Discussion

The human brain's remarkable level of specialization encompasses many functional features. For example, motor control and cognitive processes such as speech processing and action planning.^[^ ^5^ ^]^ This specialization also extends to visual processing, involving sensory detection as well as recognition of faces and objects,^[^ ^30^ ^]^ and has been shown to include asymmetries in attentional networks.^[^ ^31^ ^]^ This functional specialization is substantiated within the organizational scaffolding, which includes vertical (i.e., bottom‐up vs. top‐down information flow) as well as horizontal brain hierarchies. However, despite the significant functional role played by the horizontal connections linking the two hemispheres, much of the evidence on this phenomenon comes from studies conducted on animals.^[^ ^32^ ^]^ In humans, interest in this area has declined following the initial seminal studies on split‐brain patients.^[^ ^33^, ^34^ ^]^ This decline is surprising given the widely recognized phenomenon of hemispheric specialization in the human brain, exemplified by the established link between the left hemisphere and language,^[^ ^35^ ^]^ and the right hemisphere and attention.^[^ ^36^ ^]^ Crucially, this work has restored importance and extended this understanding by demonstrating, through a multi‐approach method—including traveling wave analysis, phase shift analysis, and Granger causality analysis—that the asymmetric organization observed in hemispheric specialization also applies to the interhemispheric V5‐V5 pathway and significantly contributes to this functional specificity in motion perception. Specifically, the study showed that individuals within the general population exhibit stronger alpha‐band Traveling Waves in the left‐to‐right pathway compared to the opposite direction. This imbalance in effective connectivity explained the tendency to have a bias toward reporting leftward motion over rightward motion in this sample. Connectivity analysis implicates posterior extrastriate circuits, classical sensory areas, as the locus of criterion modulation. This supports the view that the measured shift in c arises from changes in sensory representation of motion, rather than from motor or decisional strategy biases.

Does the highlighted evidence have an anatomical substrate that could support it? A seminal study by Genc et al.^[^ ^11^ ^]^ identified a significant association between horizontal motion perception and the microstructural integrity of corpus callosum segments interconnecting the two V5 areas. Moreover, this high correlation was topographically specific: no significant relationships were found when adjacent segments of the corpus callosum in the splenium were considered. Consistent with this, the observed imbalance in connectivity is evident only when considering the V5‐V5 connections and not the V1‐V1 connections, further suggesting the specificity of this effect to the motion‐sensitive areas. Additionally, other research has shown faster transfer of motion information across the corpus callosum from left‐to‐right compared to right‐to‐left visual cortices in humans,^[^ ^37^ ^]^ indicating enhanced access to motion information for the right V5 through facilitated transcallosal communication from the left V5. Thus, these white matter fibers could provide the structural foundation for the highlighted effective V5‐to‐V5 connectivity observed in the studies supporting conscious motion perception.

Another novel aspect raised by the study concerns the frequency‐specificity (i.e., alpha‐low beta – 9–16Hz) at which interhemispheric interaction between V5 regions occurs. There is a large amount of evidence positing that brain rhythms have a crucial role in neural information‐exchange.^[^ ^38^, ^39^, ^40^ ^]^ Specifically, previous work highlighted the crucial role of relatively low oscillations (alpha to beta) in long‐distance connectivity, whereas faster oscillations reflect rather local connectivity.^[^ ^40^, ^41^, ^42^ ^]^ Recent evidence confirms this pattern also when considering the oscillations’ phase gradient (i.e., as traveling waves).^[^ ^22^, ^23^ ^]^ It is conceivable that inter‐hemispheric interaction between motion‐sensitive areas occurred through synchronization in the alpha/low‐beta band. This empirical data matches previous computational evidence showing that brain interactions between different hierarchical levels, influenced by communication delays, produce traveling waves within the alpha band.^[^ ^19^ ^]^ Furthermore, these findings are consistent with numerous studies that have demonstrated that both stochastic^[^ ^43^, ^44^ ^]^ and voluntary modulation of alpha synchronization^[^ ^45^, ^46^ ^]^ correlate with the decision criterion adopted by participants in perceptual tasks. This further strengthens the evidence that alpha oscillations may play a crucial role in generating the subjective representation of the external world.^[^ ^47^ ^]^

When estimating propagation delays between the two motion‐sensitive areas, an average delay of ≈4 ms was obtained at the sensor level and ≈7 ms in source‐reconstructed data. However, these velocity estimates should be interpreted with caution. Scalp EEG's cortical‐source mixing attenuates and blends phase signals from different areas, compressing true temporal delays and thus making wave propagation appear faster than it actually is.^[^ ^19^ ^]^ Even source‐reconstruction methods, despite reducing mixing, still suffer from limited spatial and temporal resolution that can introduce millisecond‐scale errors into delay estimates. High‐resolution approaches, such as intracranial EEG in nonhuman primates or epilepsy patients, or local field potential recordings in animal models, will be critical for validating and refining these propagation‐velocity measurements.

While current results delineate a robust association between left‐to‐right alpha propagation and the criterion shift, a direct causal link remains to be demonstrated. A potentially promising avenue is direction‐specific cortico‐cortical paired associative stimulation (ccPAS) applied bilaterally to V5, with pulse timing that either reinforces (left‐to‐right) or counteracts (right‐to‐left) the dominant propagation direction.^[^ ^10^, ^48^ ^]^ Quantifying pre‐/post changes in travelling waves asymmetry and criterion after each protocol will reveal whether boosting interhemispheric connectivity amplifies the bias and, conversely, whether reversing the temporal order attenuates it, thereby testing whether causal manipulation of wave propagation underpins the behavioral effect.

Moreover, while current findings reveal a left‐to‐right propagation bias, further studies are needed to assess whether this asymmetry is hardwired or experience‐dependent. For instance, comparing readers who habitually read from right to left (e.g., Arabic readers)^[^ ^49^, ^50^ ^]^ could clarify whether directional propagation and perceptual bias can be modulated by long‐term habits. Similarly, developmental studies could test whether experience‐driven plasticity amplifies these dynamics with age. The observed asymmetry may reflect the distinct functional roles of the two hemispheres in sensory analysis, with the left hemisphere prioritizing perceptual details and the right integrating information into a more holistic representation.^[^ ^51^, ^52^, ^53^, ^54^ ^]^ These interactions can be interpreted within the framework of evidence accumulation‐to‐bound models,^[^ ^55^ ^]^ in which the left hemisphere may transmit discrete sensory evidence to the right hemisphere for integration into a coherent percept. Supporting this interpretation, Zhang et al.^[^ ^56^ ^]^ showed that alpha oscillations contribute to feature binding in visual perception, facilitating the integration of color and motion cues. Accordingly, the increased alpha synchronisation observed between left and right V5 may facilitate the construction of a unified motion percept.

This line of interpretation fits with some evidence deriving from the clinical population. For example, it is well recognized that individuals with autism often exhibit a “piece‐to‐piece” perception style, reflecting a deficiency in information integration.^[^ ^57^, ^58^, ^59^, ^60^, ^61^, ^62^, ^63^ ^]^ As a result, individuals with autism needed stronger facilitation to experience horizontal motion in the motion quartet.^[^ ^64^ ^]^ According to this evidence, it is conceivable that autistic individuals would demonstrate less leftward bias in the RDM task. This could be attributed to hypo‐communication between brain areas, potentially resulting in reduced strength of cross‐hemispheric traveling waves.

Additionally, human psychophysical experiments have shown that priming participants with a brief exposure to real motion significantly increases the likelihood of perceiving motion in an illusory context in the congruent direction.^[^ ^65^ ^]^ This effect could rely on the pre‐activation of alpha/low beta traveling waves congruent with the primed direction. This prompts further investigation, as it could be explored whether directional priors about upcoming movement^[^ ^66^ ^]^ could bias perception through concurrent modulation of V5‐V5 connectivity. For instance, it could be hypothesized that prior expectations of leftward movement strongly activate Left‐to‐Right V5 traveling waves, resulting in a stronger leftward bias.

## 4 Conclusion

In summary, the pathway between human left and right V5s exhibits a hierarchical organization, with left‐to‐right connections being overrepresented compared to the flow in the opposite direction. This imbalance predicts the subjective experience of motion directionality in the RDM paradigm: the greater the imbalance between the two information flows, the stronger the participant's leftward bias in motion perception. Crucially, unlike previous findings that demonstrated this tendency in illusory contexts,^[^ ^8^ ^]^ this study outlined that this bias is also present in tasks like RDM where a specific motion direction (i.e., right vs. left) is objectively present on a trial‐by‐trial basis. This implies that this tendency is so strong that it is able to override objective sensory evidence. Furthermore, it was identified the rhythmic code underlying these interhemispheric interactions, implicating alpha/low beta waves. This evidence supports and reinforces previous studies linking alpha/low beta frequency bands to visual binding,^[^ ^56^ ^]^ perceptual outcomes,^[^ ^67^, ^68^, ^69^, ^70^ ^]^ and decisional bias.^[^ ^45^, ^46^ ^]^ Collectively, these findings offer new mechanistic insights into how hierarchical models of neural networks operate within the human brain.

## 5 Experimental Section

### Participants

42 right‐handed participants (26 female, age range 18–30) with normal or corrected‐to‐normal visual acuity took part in the study. Before starting the experiment, they gave informed consent, and all the experimental procedures were performed in accordance with the Declaration of Helsinki.

### Experimental Protocol

Visual stimuli consisted of 400 white dots moving within a square region at the centre of the screen and were presented at a distance of 70 cm while participants sat comfortably in a dimly lit room. Stimuli were generated and presented using Matlab (version 2016a) and the Psychophysics toolbox.^[^ ^71^ ^]^ In a preliminary phase, participants performed a brief and simplified demo version of the task with stimuli presented for 1000s, and then a training phase in order to familiarize with the task. Then, participants underwent a titration procedure to determine the coherence of movement for which accuracy was 75%. The main task consisted of 216 trials. Within each trial, the titrated percentage of dots could move either leftward or rightward, and participants were required to indicate the perceived direction of movement by pressing a key on the keyboard (e.g., the left arrow key to signal left movement) as quickly and accurately as possible. Each dot moved at a speed of 4.5°/second.

### Signal‐Detection Theory (SDT) Modeling

SDT measures d’ and c^[^ ^12^ ^]^ were computed. d’ quantifies a participant's stimulus sensitivity (higher d’ values are indicative of higher sensitivity), whereas c quantifies a subject's decision criterion (c value different from 0 implies the presence of choice bias). These measures were calculated based on the proportion of hits (i.e., reporting right movement in rightward‐moving trials) and false alarms (i.e., reporting right movement in leftward‐moving trials) using these formulas:

(1) $$d^{\prime }=Z\left(Hit\;Rate\right)-Z\left(False\;Alarm\;Rate\right)$$

(2) $$c=-\frac{Z\left(Hit\;Rate\right)+Z\left(False\;Alarm\;Rate\right)}{2}$$

where Z(x) represents the z‐score transformation (the inverse function of the standard normal cumulative distribution). While c is formally a response bias,^[^ ^12^ ^]^ criterion shifts can originate from changes in sensory encoding.^[^ ^43^, ^47^ ^]^ Given the posterior alpha phase asymmetry observed here, the measured shift in c was treated as indicative of a perceptual bias in motion representation. Then, It was evaluated whether there was a bias in perceiving a specific direction of movement. To this end, a t‐test against zero was conducted to assess whether the decision criterion was significantly higher (indicating a bias toward the left) or lower (indicating a bias toward the right) than zero. All descriptive values in the manuscript are reported as mean ± SEM.

### EEG Acquisition and Preprocessing

Participants were comfortably seated in a room, while a set of 64 electrodes adhered to the international 10–10 system was set up. EEG signals were acquired at a rate of 1000Hz, with all impedances maintained below 10kΩ. Subsequently, offline processing of EEG data was conducted using custom MATLAB scripts (version R2021a) and the EEGLAB toolbox.^[^ ^72^ ^]^ Offline filtering of EEG recording was implemented in the 0.5–125Hz band. To remove 50 Hz line noise, the Cleanline plugin from the EEGLAB toolbox was applied.^[^ ^72^ ^]^ Visual inspection of signals was carried out, and any noisy channels were spherically interpolated. Epochs spanning ‐1.5 to 1 s relative to the stimulus onset were extracted, and individual trials were visually reviewed, with those exhibiting excessive noise and muscle artifacts being discarded. After this step, an average of 183.31 ± 3.43 epochs remained per participant. Next, the recording was re‐referenced to the average of all electrodes, and Independent Component Analysis (ICA), an effective and widely employed method for EEG artifact removal, was applied. Components containing artifacts distinguishable from brain‐driven EEG signals (mean 3.95 ± 0.37) were subtracted from the data. Following these procedures, the signals were downsampled to 256Hz.

### Traveling Waves Analysis

**Figure 4:**
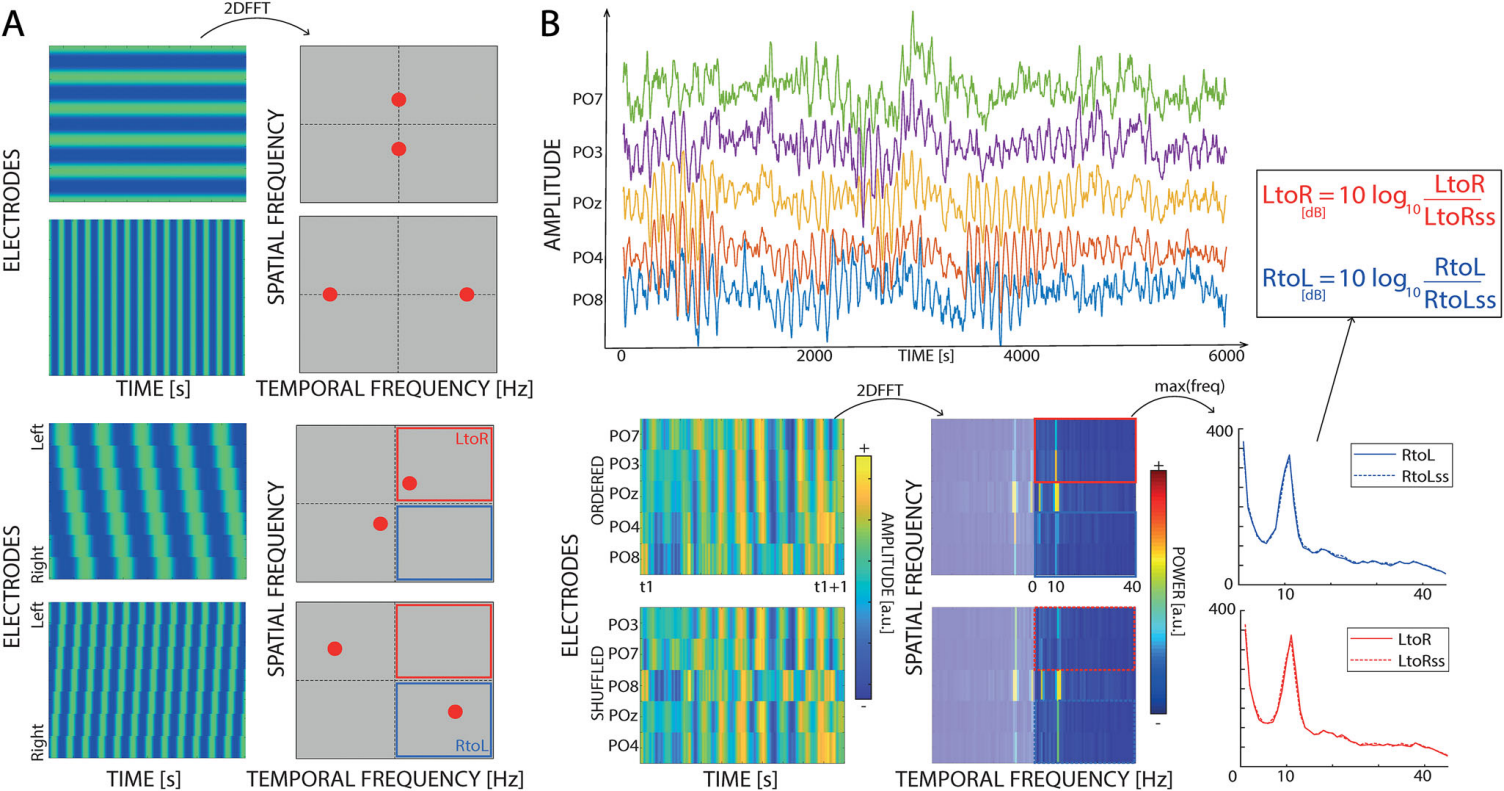
Waves analysis. A) Images, having space (i.e., electrodes) and time as axis, are decomposed using 2D‐Fast Fourier Transform (2D‐FFT). Considering 2D sinusoid propagating along the vertical or horizontal axis of the image, the 2D‐FFT returns spectral peaks on one of the two axis, depending on the direction of propagation, and their position on the axis depends on the frequency of the oscillations (as in the 1D‐FFT). When the oscillations propagate with a positive or negative phase shift, the spectra change accordingly. Importantly, the spectral peaks provide a reliable measure to differentiate waves propagating in either direction. B) First, images were defined over a time window, and then a 2D Fourier transformation was computed to quantify the amount of RtoL (in blue) and LtoR (in red) waves. From each quadrant, the maximum value over spatial frequencies was extracted and the analysis focused over the 2–45Hz band. The same procedure was performed after shuffling the electrodes’ order to obtain a surrogate measure, which was used as a baseline. Importantly, such a measure acts as a null distribution, capturing (and removing) the 1/f trend and the intrinsic features of the spectrum that are not related to the spatial component of the signal (i.e., non‐traveling components).

Traveling waves’ propagation was quantified along a line of 5 electrodes, running from the left and the right hemisphere. As illustrated in Figure 1, the crucial line of electrodes included those covering the homologous motion areas. Previous research has shown that PO7 and PO8 were situated closest to the human visual areas.^[^ ^16^, ^17^ ^]^ Therefore, to investigate the inter‐hemispheric interaction between these regions, the line of electrodes connecting them (PO7, PO3, PZ, PO4, PO8) was selected. Additionally, other lines of electrodes spanning both hemispheres were chosen to serve as a control (centro‐parietal line: CP3‐CP1‐CZ‐CP2‐CP4; frontal line: AF7, AF3, AFZ, AF4, AF8). For each set of five electrodes, 2D maps were created by sliding a 500 ms time window over the EEG signals (having a 125 ms overlap) and computing 2D‐FFT representations of each map (**Figure** **4**A). Notably, the power in the upper and lower quadrants quantifies the amount of waves propagating in the left‐to‐right (LtoR – from left to right hemisphere) and right‐to‐left (RtoL – from right to left hemisphere) direction, respectively (Figure 4B). Next, the same procedure was performed after shuffling the electrodes to obtain a baseline with the same spectral power but without spatial information about the amount of L‐to‐R and R‐to‐L waves. This shuffling procedure provides a baseline that quantifies the presence of a traveling wave in each time window. Lastly, for each frequency in the range 2–45 Hz, the maximum values in the 2D‐FFT spectra were extracted in both the real (LtoR and RtoL) and the shuffled data (LtoRss and RtoLss), obtaining the waves’ amount in decibel [dB] as:

(3) $$LtoR_{waves}\left[dB\right]=10*\left(\frac{LtoR}{LtoR_{SS}}\right)\;$$

(4) $$RtoL_{waves}\left[dB\right]=10*\left(\frac{RtoL}{RtoL_{SS}}\right)\;$$

Then, It was investigated whether the amount of prestimulus (‐400 to 0 ms) left‐to‐right (LtoR) and right‐to‐left (RtoL) waves exhibited different magnitudes. A data‐driven approach was adopted to select the frequency band for analysis. Specifically, the average time‐frequency map of traveling waves was considered, regardless of the direction of connectivity (i.e., averaging left‐to‐right and right‐to‐left maps). This approach ensured an unbiased analysis as the waves were averaged irrespective of connectivity direction. The result revealed that inter‐hemispheric interaction predominantly occurred within the alpha‐low beta band (9‐16Hz). It was then examined whether the magnitude of traveling waves in this frequency band differed between the LtoR and RtoL directions. Additionally, it was investigated whether this effect was specific to electrodes over the motion areas by analysing whether a similar inter‐hemispheric imbalance was present in anterior electrodes. Moreover, It was controlled that the highlighted effect was frequency‐specific when considering the theta (5‐8Hz) and high‐beta (17‐25Hz) bands. Additionally, it was examined whether the local effect resulting from differences in power could account for the observed disparities in traveling waves. To investigate this, power within the alpha‐low beta band was extracted from electrodes PO7, PO3, PO4, and PO8, and conducted paired t‐tests between PO7 and PO8, as well as between PO3 and PO4, to discern any discrepancies. Furthermore, when analyzing traveling waves in EEG signals, it was important to consider significant limitations related to the difference between waves propagating in the cortex and waves measured at the sensor levels.^[^ ^73^ ^]^ This includes artifacts related to the disruption due to long‐range connections, as well as signal smearing caused by scalp interference.^[^ ^74^, ^75^, ^76^ ^]^

### Brain‐2‐Behavioural Analysis

It was assessed whether the hemispheric imbalance in alpha/low‐beta traveling wave connectivity could predict the magnitude of individual biases in motion perception. To this end, Pearson and Spearman correlations were computed between each participant's wave laterality index and their perceptual criterion. The wave laterality index was calculated as the difference in wave strength between the LtoR V5 direction and the RtoL V5 direction. Positive values indicate greater wave propagation from left to right V5, compared to the opposite direction. This index, computed at the individual level, was then correlated with the criterion derived in the previous analysis, where negative values indicate a bias toward leftward motion. Accordingly, a negative relationship between the two measures was hypothesized: the stronger the relative left‐to‐right wave propagation (compared to right‐to‐left), the greater the bias toward leftward motion.

To investigate the robustness of the result, robust correlations were also conducted using the robust correlation toolbox,^[^ ^77^ ^]^ which eliminates any outliers that may influence the correlation.It was explored whether the imbalance in interhemispheric communication was related to sensitivity in the task as well. In addition, participants were categorised based on whether they had a greater flow going from PO7 to PO8 versus those with a greater flow from PO8 to PO7 and assessed whether the two groups exhibited different decisional biases.

### Phase‐Shift Analysis

To analyze the phase shift between the PO7 and PO8 electrodes in the alpha‐beta frequency band (9–16 Hz), a band‐pass filter was applied to isolate the frequency range of interest. The filtering was performed using a finite impulse response (FIR) filter with a transition width of 15%. For each trial, the instantaneous phase of both electrodes was extracted using the Hilbert transform. The phase shift was then computed by subtracting the phase of PO8 from that of PO7, resulting in a trial‐specific phase difference, which was treated as a complex vector with unit magnitude. These complex vectors were summed across trials for each participant to calculate a circular mean phase shift, which provides a robust estimate of the overall phase shift between the two electrodes. Then, the Rayleigh test for non‐uniformity was employed to statistically assess the significance of the observed phase shift across participants. A significant result would indicate that the phase differences between the two electrodes were not uniformly distributed, implying a consistent phase relationship across trials in the alpha‐beta band. Moreover, to corroborate the rightward (left‐to‐right) posterior propagation inferred from the primary PO7‐to‐PO8 phase analysis, the sensor‐level comparison was extended to the intermediate right‐hemisphere neighbours of PO7: PO3, POz, and PO4 (in addition to PO8). Using the identical preprocessing, alpha‐band extraction, and instantaneous phase estimation procedures described for the PO7‐to‐PO8 analysis, PO7‐to‐X phase differences (phase_PO7 – phase X) were computed for each participant and tested them using the Rayleigh test for nonuniformity. The same phase extraction approach was then applied in source space (see below) to the left and right V5 (MT+) estimates in order to test whether the same pattern held valid when considering the activity at the source level. Finally, to ensure temporal alignment across analyses, the time window for this phase shift analysis was restricted to the same interval used in the traveling wave analyses (–400 to 0 ms relative to target onset).

### Granger Causality Analysis

To determine the degree of causal connectivity within the V5‐V5 network using a complementary approach to the traveling waves, Granger causality analysis was used as implemented in the Multivariate Granger Causality Toolbox (MVGC),^[^ ^78^ ^]^ which implements Granger causality analysis based on vector autoregressive (VAR) modeling—fitting each electrode's signal as a combination of its own past activity and that of the other electrode, thus capturing the temporal influence one region exerts over the other. As Granger causality analysis relies on vector autoregressive (VAR) modeling, it requires that the input signals satisfy the assumption of stationarity. To meet this requirement, linear trends were removed from each trial by fitting and subtracting a first‐order polynomial (using MATLAB's polyfit functions). This step was mandatory to prevent slow drifts or baseline fluctuations—unrelated to true neural interactions—from introducing spurious temporal dependencies that can bias Granger causality estimates. The model order was selected in a data‐driven manner using the Akaike Information Criterion (AIC), setting the maximum allowable order to 6 in order to match the temporal window adopted in previous studies.^[^ ^62^, ^63^, ^79^, ^80^ ^]^ The AIC evaluates model quality by balancing goodness of fit and model complexity, favoring models that explain the data well while minimizing the risk of overfitting. The model order refers to the number of past time points (lags) included in the Vector Autoregressive (VAR) model used for estimating Granger causality. All epochs were retained, and focused on the time period from ‐400 to 0 ms relative to the target onset for computing the Granger indices. in order to align this analysis with the time window used for the traveling wave analysis. This alignment ensures that both analyses capture the same prestimulus neural dynamics, enhancing the interpretability and consistency of the results. Based on the resulting parameters, Granger causality was computed in the frequency domain. The resulting values were then averaged between 9–16 Hz, considering the PO7‐to‐PO8 and PO8‐to‐PO7 directions separately. To ensure robust group‐level estimates, outlier participants (N = 1) whose Granger values deviated by more than ± 4 standard deviations from the group mean were identified and excluded. To ascertain whether the causality index was stronger from the left‐to‐right direction compared to the right‐to‐left direction, a paired sample t‐test was conducted. As a further control analysis, the same procedure was repeated for the 5–8 Hz (theta) and 17–25 Hz (high‐beta) frequency bands to check the frequency‐specific nature of the effect. Crucially, these control analyses found no differences in Granger causality when considering these frequency bands (t_40_ < 1.63, all p > 0.11, all BF < 0.57).

### Cortical Source Reconstruction

**Figure 5:**
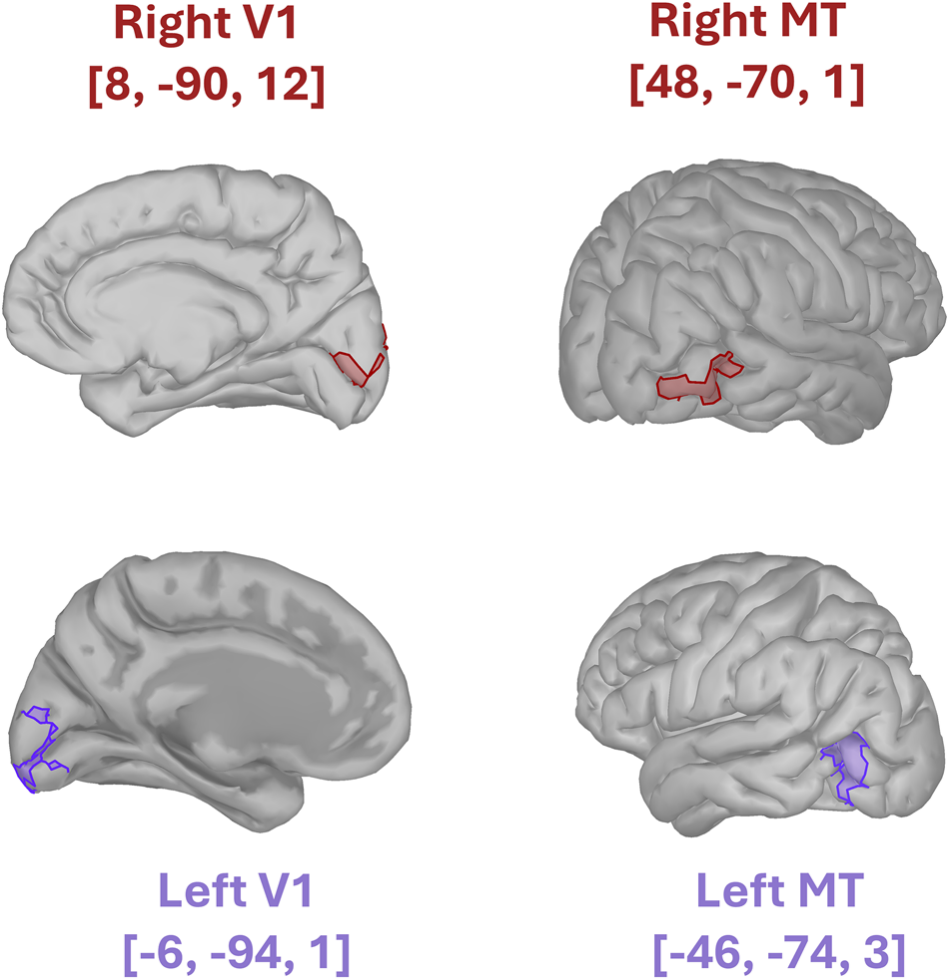
Cortical source reconstruction. Cortical source activity was reconstructed from pre‐processed EEG signals to extract four regions of interest (ROIs): left MT, right MT, left V1, and right V1. This process utilized the Brainstorm toolbox^[^ ^81^ ^]^ and employed a three‐layer template head model based on the ICBM152 MNI template, including the scalp, outer skull surface, and inner skull surface. The model featured a discretized cortical source space with 15,002 vertices, implemented via the Boundary Element Method in OpenMEEG.^[^ ^82^ ^]^ For estimating cortical sources, the standardized Low‐Resolution Electromagnetic Tomography (sLORETA) algorithm^[^ ^83^ ^]^ providing a linear inverse solution with zero dipole‐localization error under ideal conditions. The ROIs were identified according to the Brainnettome atlas:^[^ ^84^ ^]^ MNI _left MT_ = [‐46, ‐74, 3]; MNI _right MT_ = [48, ‐70, ‐1]; MNI _Left V1_ = [‐6, ‐94, 1]; MNI _Right V1_ = [8, ‐90, 12] .

The very same analysis was then repeated, this time at the source level. To this end, cortical source activity was reconstructed (**Figure** **5**) from the pre‐processed EEG signals using the Brainstorm toolbox.^[^ ^81^ ^]^ A three‐layer template head model, consisting of the scalp, outer skull surface, and inner skull surface (ICBM152 MNI template) was utilized, to address the forward problem. This model included a discretized cortical source space with 15, 002 vertices and was implemented using the Boundary Element Method in the OpenMEEG software.^[^ ^82^ ^]^ For the estimation of cortical sources, the standardized Low‐Resolution Electromagnetic Tomography (sLORETA) algorithm^[^ ^83^ ^]^ was adopted. This method was a linear inverse solution technique designed for modeling 3D distributed EEG sources. sLORETA computes a weighted minimum norm solution, relying on standardized current density estimates for localization inference. The resulting solution was instantaneous, distributed, discrete, and linear, exhibiting zero dipole‐localization error under ideal (noise‐free) conditions. The accuracy of our model was ensured by using constrained dipole orientations, with each dipole oriented perpendicularly to the cortical surface. Finally, the cortical sources were grouped into four distinct regions of interest (ROIs) based on the Brainnetome atlas^[^ ^84^ ^]^ available within the Brainstorm toolbox: left MT, right MT, left V1, and right V1 (MNI _left MT_ = [‐46, ‐74, 3]; MNI _right MT_ = [48, ‐70, ‐1]; MNI _Left V1_ = [‐6, ‐94, 1]; MNI _Right V1_ = [8, ‐90, 12]). Scout time series were then extracted from these 4 ROIs. For the Granger causality analysis, the MVGC toolbox was employed on these source‐reconstructed scout time series. It was then evaluated whether the Granger causality between left and right MT, as well as between left and right V1, exhibited distinct directional patterns.

## Conflict of Interest

The authors declare no conflict of interest.

## Supporting information



Supporting Information

## Data Availability

The data that support the findings of this study are available from the corresponding author upon reasonable request.
