# Supplementary material for: Perceptual Bias in Motion Discrimination is Related to Asymmetric Interhemispheric Alpha Traveling Waves
Source: Adv Sci (Weinh). 2025 Aug 30;12(41):e14623. doi: 10.1002/advs.202414623 (PMC12591206; doi:10.1002/advs.202414623)
Supplement: Supplementary file 1 — Supporting Information [file ADVS-12-e14623-s001.docx]

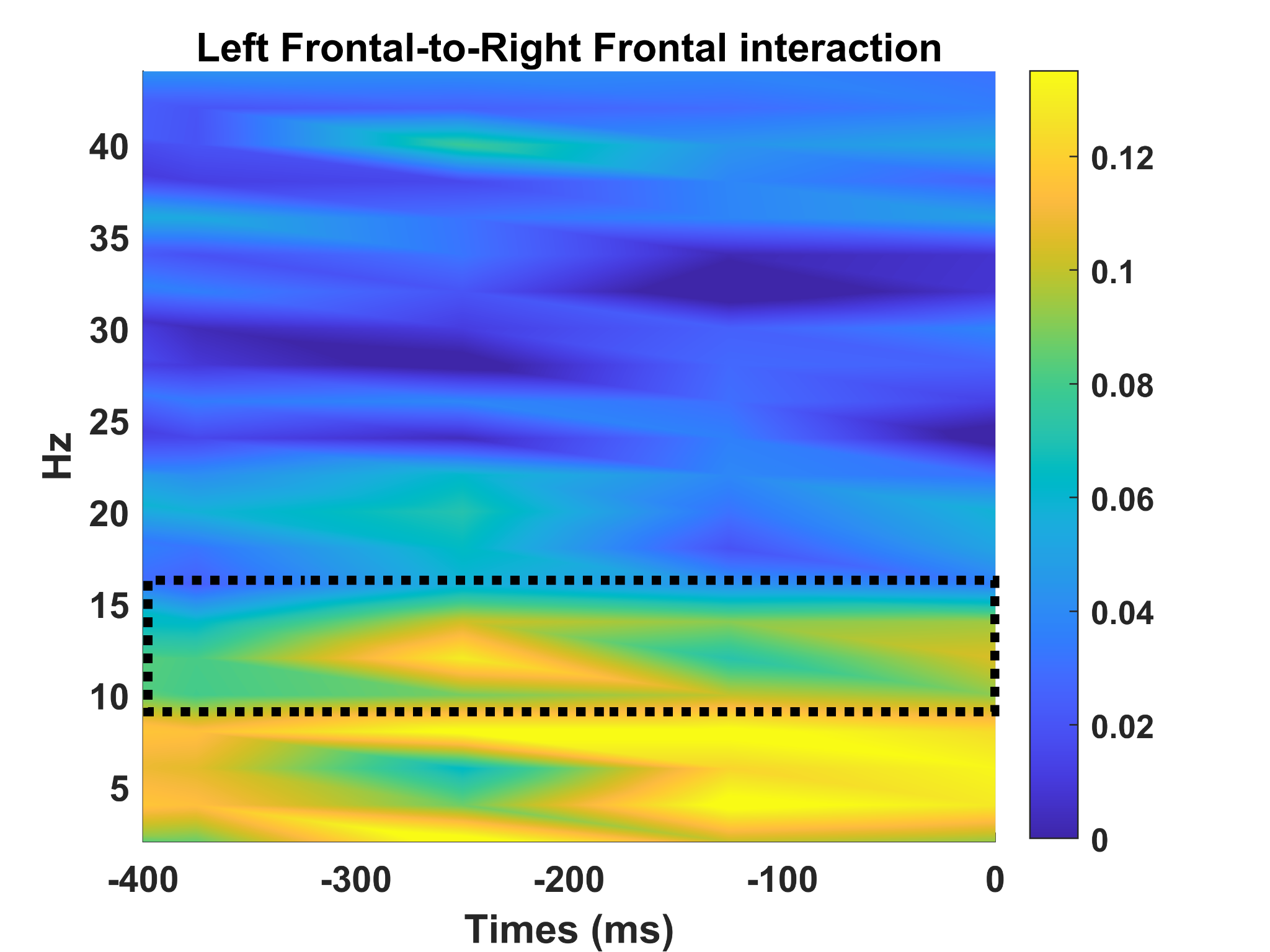

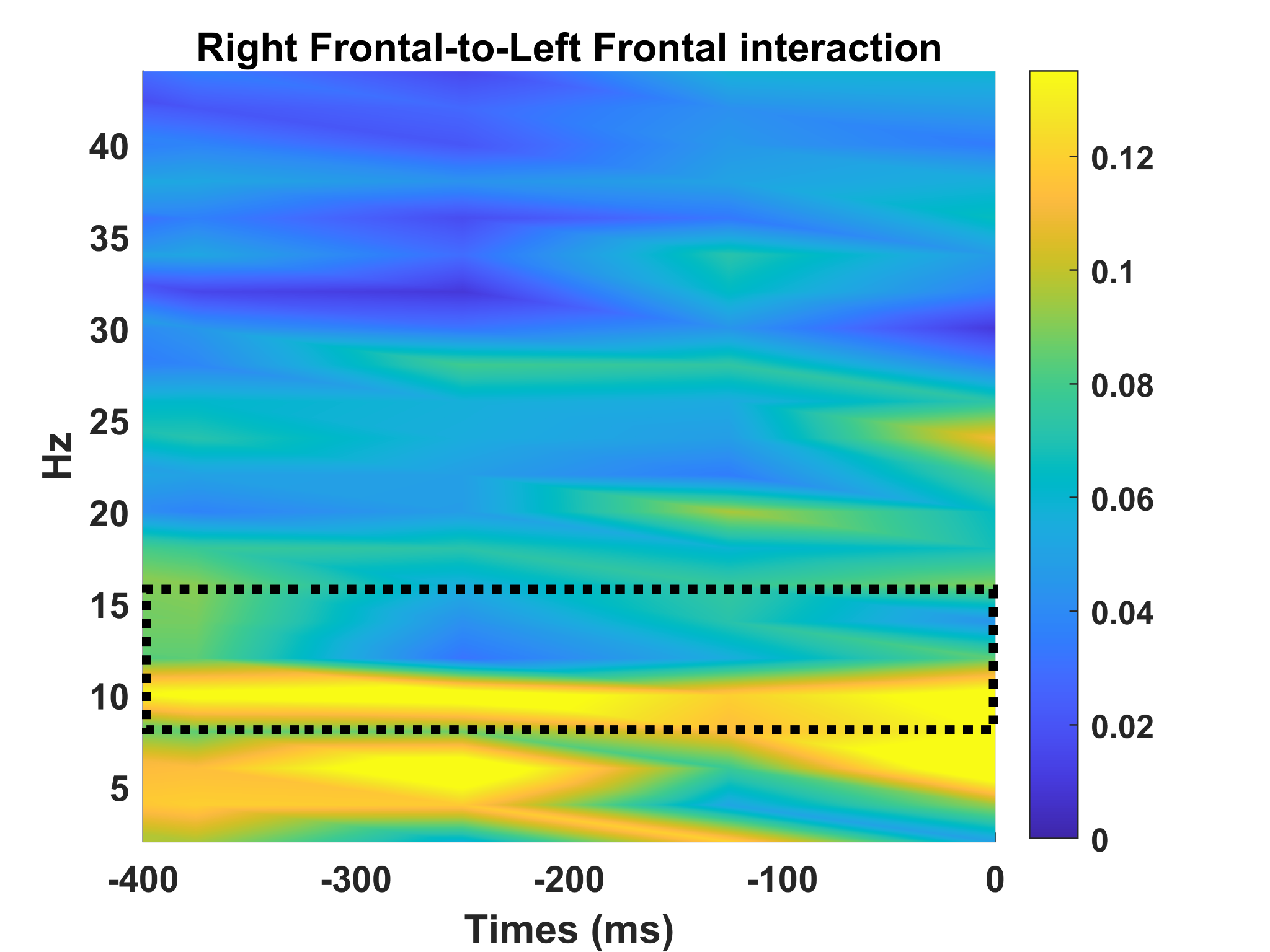


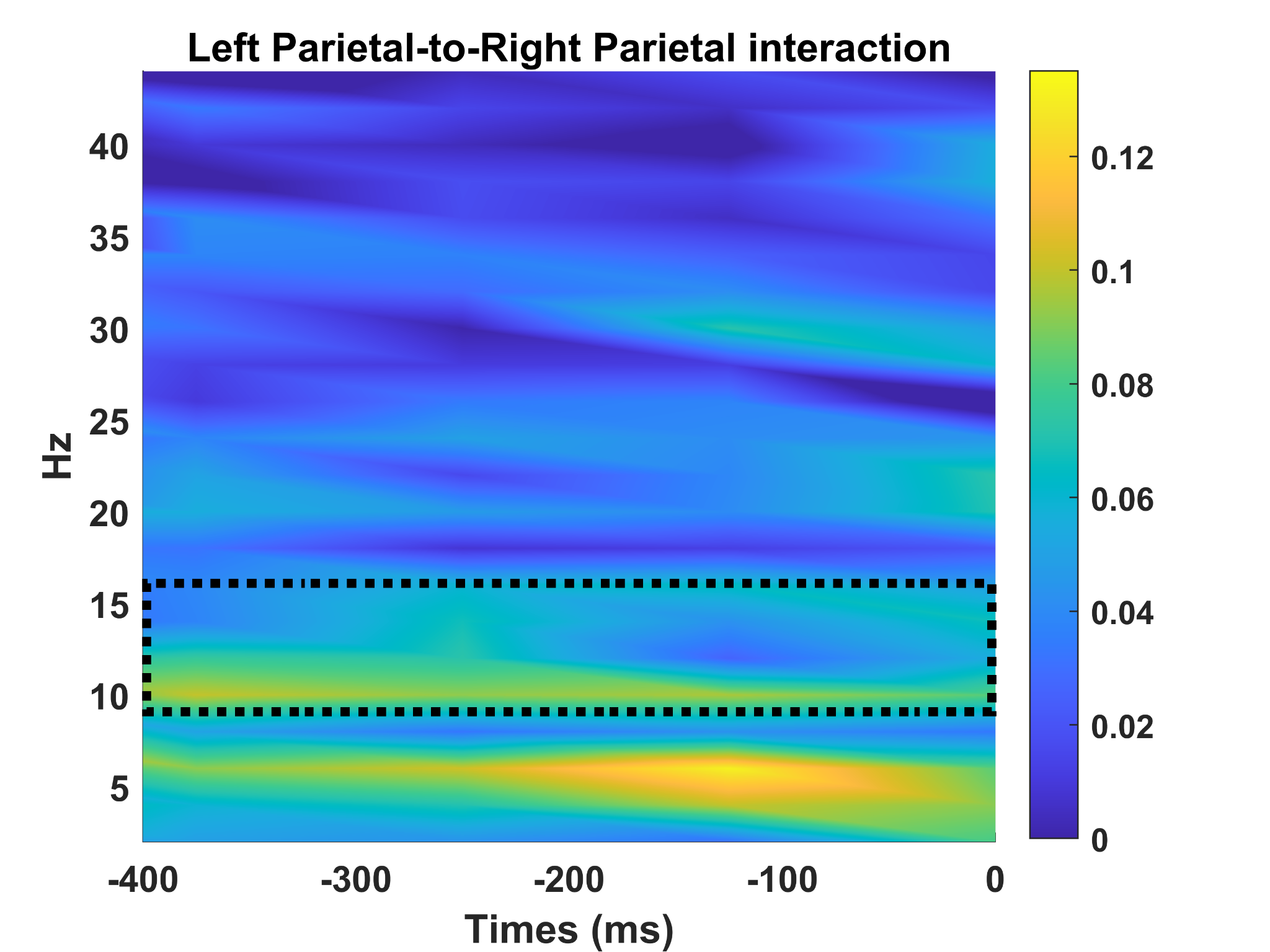

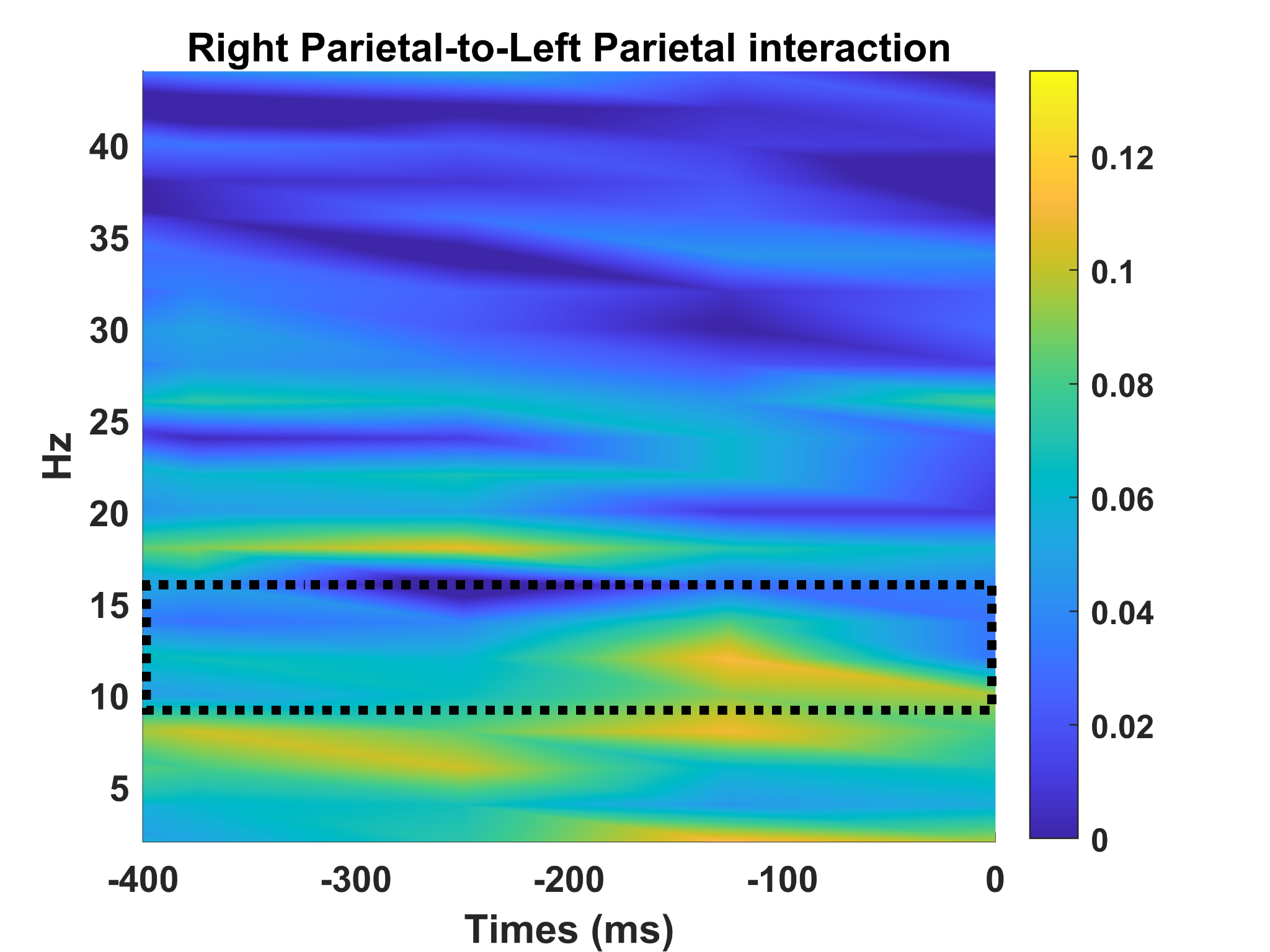


**Figure S1**

In the main text, we demonstrated a strong divergence when considering the directionality of connectivity in the V5-V5 network, with a pronounced emphasis on left-to-right direction compared to right-to-left. Crucially, this effect was specific to the visual system, as the same interhemispheric imbalance in traveling wave strength was not identified within the centro-parietal and fronto-frontal interhemispheric connections (all t_41_ < 0.94, all p > 0.35).


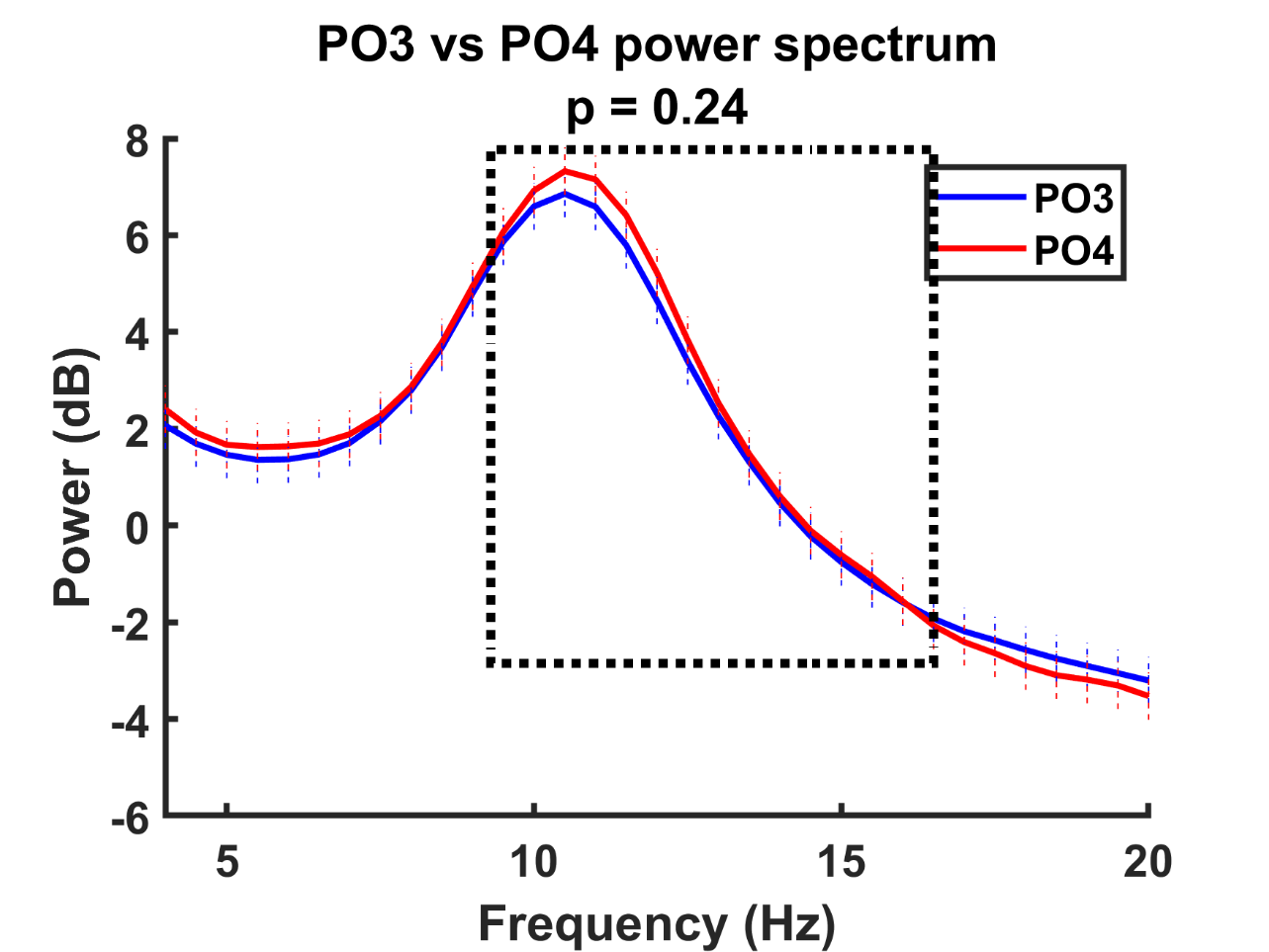


**Figure S2**

In the main text, we showed that the imbalance in traveling waves observed in the V5-V5 network was not reflected in a similar imbalance at the local level, as the power computed in the PO8 and PO7 electrodes did not differ significantly. Here, we corroborate the same analysis when comparing PO3 and PO4 (t_41_ = -1.05, p = 0.30).

**
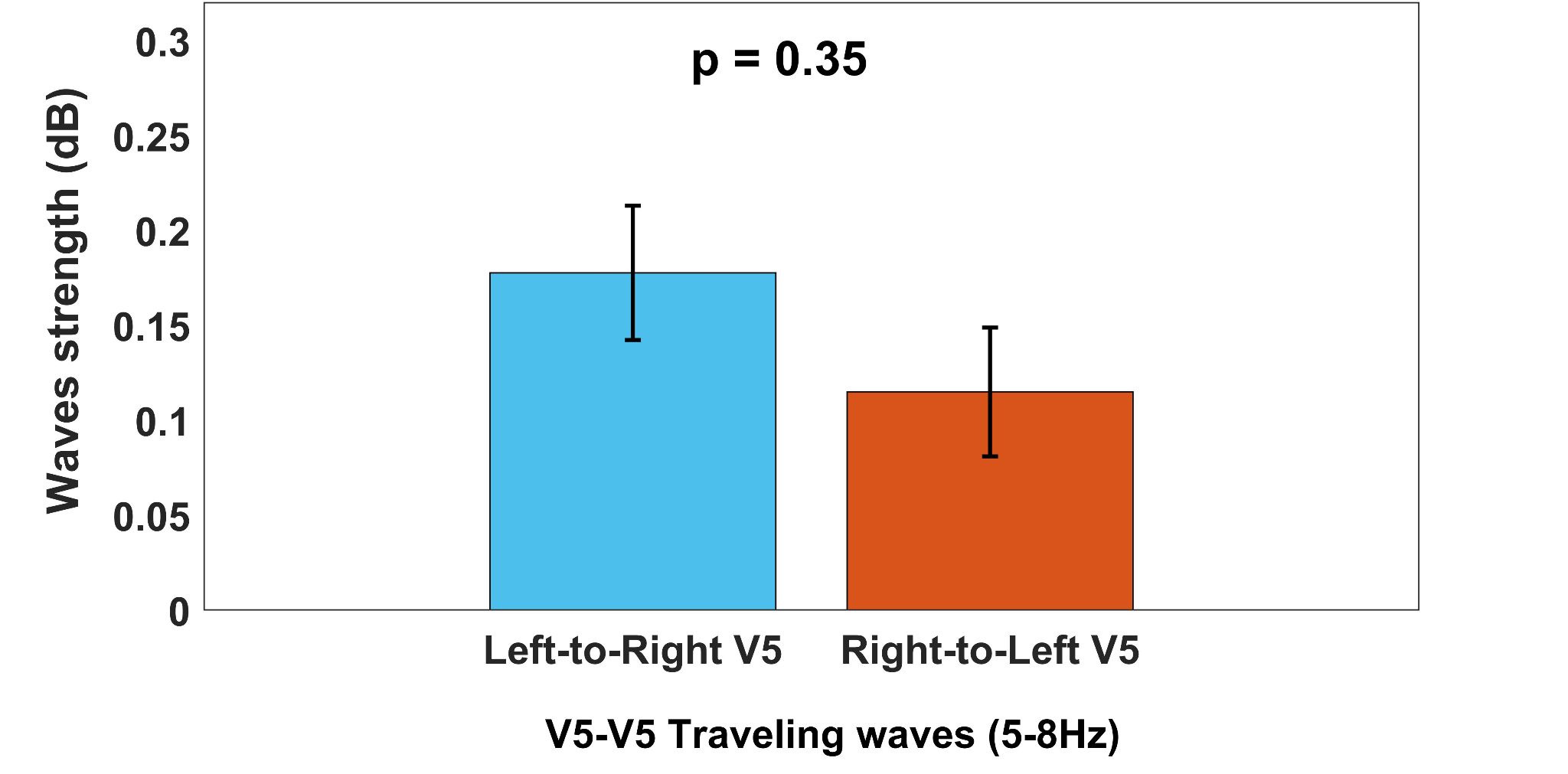
**

**
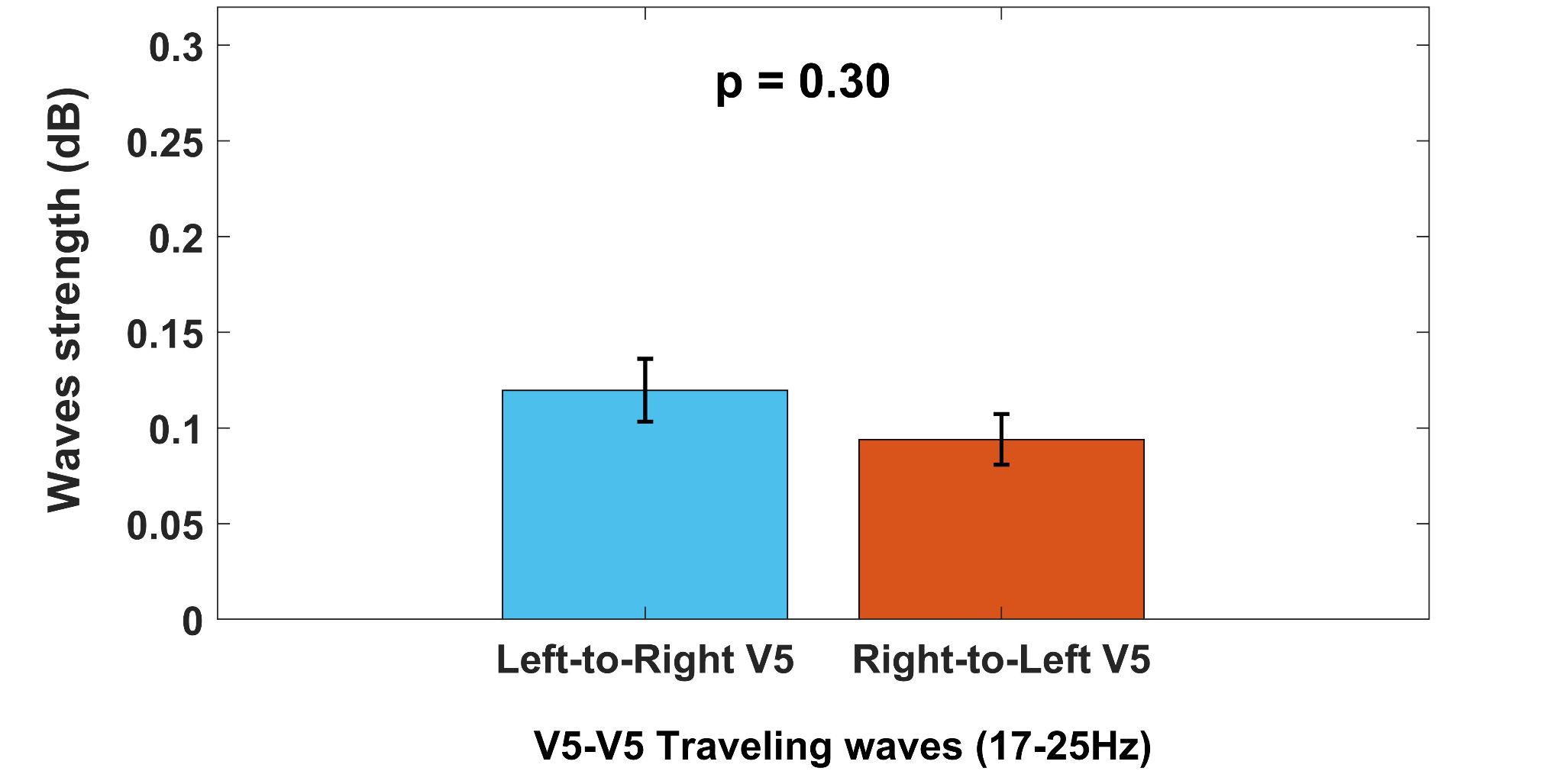
**

**Figure S3**

In the main text, we showed a stronger left-to-right flow in the alpha-beta band considering the V5-V5 network. Control analysis demonstrated that the effect was frequency-specific because there was no difference in the traveling wave strength in the theta (5-8Hz) and higher-beta bands (17-25Hz) based on the directionality of the connections considered (all t_41_ < 1.04, all p >0.30).
